# Robotic-assisted virtual care

**DOI:** 10.1093/geroni/igab046.3100

**Published:** 2021-12-17

**Authors:** Lillian Hung

**Affiliations:** University of British Columbia, Vancouver, British Columbia, Canada

## Abstract

Heightened isolation during the pandemic has exacerbated the stress, anxiety, and adverse consequences through the loss of family connections older people experience in LTC. Heavy workload and staffing shortage limit staff’s capacity to assist residents in accessing regular virtual visits. Using a Collaborative Action Research (CAR) approach, this project aims to assess the implementation of a telepresence robot, Double 3 to help residents connect with their families. CAR allows careful planning of implementation with stakeholders (patient and family partners, staff, and decision-makers), tailoring adaption to the complex LTC environment. We will program path planning to allow efficient movement between target destinations (residents' rooms) and the charging dock. For example, the robot will go to a resident’s room every morning or evening to help the resident to make a virtual call with family. The project involves three phases (a) Observe and Reflect, (b) Act and Adapt, (c) Evaluate. We work with two Canadian LTC homes in British Columbia to investigate feasibility and acceptability. CAR emphasizes research with, rather than research on people. Meaningful engagement with patient and family partners, frontline staff, and decision-makers at each site throughout the whole project will ensure the project will meet the local needs. Anticipated resident outcomes include improved quality of life, mood, perceived loneliness, perceived social support, and acceptance. Anticipated staff outcomes include perceived ease of use, and acceptability.

